# Automated determination of 8-OHdG in cells and tissue via immunofluorescence using a specially created antibody

**DOI:** 10.1016/j.btre.2024.e00833

**Published:** 2024-03-02

**Authors:** Tobias Jung, Nicole Findik, Bianca Hartmann, Katja Hanack, Kai Grossmann, Dirk Roggenbuck, Marc Wegmann, René Mantke, Markus Deckert, Tilman Grune

**Affiliations:** a Department of Molecular Toxicology, German Institute of Human Nutrition Potsdam-Rehbruecke (DIfE), Arthur-Scheunert-Allee 114-116 14558, Nuthetal, Germany; b German Center for Cardiovascular Research (DZHK), 10117, Berlin, Germany; c German Center for Diabetes Research (DZD) 85764 Muenchen-Neuherberg, Germany; d NutriAct – Competence Cluster Nutrition Research Berlin-Potsdam, 14558 Nuthetal, Germany; e University of Potsdam, Institute of Nutrition 14558 Nuthetal, Germany; f GA Generic Assays GmbH, Germany; g Faculty of Health Sciences Brandenburg, Brandenburg Technical University Cottbus-Senftenberg; h new/era/mabs GmbH, August-Bebel-Str. 89 14482 Potsdam, Germany; i University of Potsdam, Department of Biochemistry and Biology, Chair of Immunotechnology, Karl-Liebknecht-Str. 24-25, Build 29, Office 1.55 14476 Potsdam, Germany; j Brandenburg Medical School Theodor Fontane, Klinik für Allgemein- und Viszeralchirurgie, Klinikum Brandenburg, Hochstraße 29 14770 Brandenburg an der Havel, Germany; k Brandenburg Medical School Theodor Fontane, Theodor Fontane Campus Brandenburg, Hämatologie, Onkologie SKB, IAG Psychoonkologie und Palliativversorgung, Hochstraße 29, 14770 Brandenburg an der Havel, Germany; l MEDIPAN GmbH, Ludwig-Erhard-Ring 3 15827 Dahlewitz

**Keywords:** 8-OHdG, DNA-damage, Immunostaining, Histology, Cancer

## Abstract

Despite powerful DNA repair systems, oxidative damage/modification to DNA is an inevitable side effect of metabolism, ionizing radiation, lifestyle habits, inflammatory pathologies such as type-2 diabetes or metabolic syndrome, cancer and natural aging.

One of the most common oxidative DNA modifications is 8-OHdG (8‑hydroxy-2′-deoxyguanosine), which is the most widely used marker in research and clinical diagnostics. 8-OHdG is easily and specifically detectable in various samples such as urine, plasma, cells and tissues via a large variety of methods like ELISA, HPLC, chromatographic methods, and immunochemistry.

Formed by oxidation of guanine and being representative for the degree of DNA damage, 8-OHdG can be also used as biomarker for risk assessment of various cancers as well as degenerative diseases.

Here, we present a highly specific, self-developed 8-OHdG antibody in successful comparison to a commercially one, tested in cells (FF95, HCT116, and HT22) and intestinal tissue, focusing on automatized evaluation via fluorescence/confocal microscopy.

## Highlights

• Here, a new 8-OHdG-specific antibody is presented.
• The antibody functions in both cells and tissues.
• It is qualitatively and quantitatively representative for 8-OHdG.
• And is therefore suitable for fully automated, high-throughput processing.

## 1 Introduction

Permanent formation of reactive oxygen (ROS) and nitrogen species (RNS) is an inevitable side-product fund in all living (not only) anaerobic cells, due to the chemical processes necessary for metabolism and cellular functionality[1], [2], [3], [4]. Reactive species are able to damage/oxidatively modify virtually any organic cell compound like proteins[5], [6], [7], [8], [9], lipids (peroxidation)[9], [10], [11], [12], sugars and also nucleobases as present in RNA and DNA[2,3,13,14].

Under normal physiological conditions, there is a robust balance between ROS formation and the cellular antioxidative machinery, that includes low molecular antioxidants, enzymatic antioxidants and also powerful systems, that are able to repair already induced damage[15,16]. Furthermore, there is also a large variety of systems and strategies, able to restore oxidatively damaged DNA like DNA-polymerases[17,18] (recognize and replace damaged bases). The main DNA repair pathways are nucleotide excision repair (removes specific types of DNA damage, including UV-induced pyrimidine dimers and chemically induced damage such as bulky adducts)[19], base excision repair (removes damaged bases and replaces them with new, correct bases)[20], mismatch repair (recognizes and corrects errors that occur during DNA replication, including mismatches of base pairs)[21,22], homologous recombination (damaged DNA is repaired using intact DNA material as a template, often used to repair double-strand breaks)[23] and non-homologous end joining (repairs double-strand breaks; in this case, the ends of the broken strand are directly joined together without using homologous DNA as a template)[24,25].

If an imbalance between damage and repair occurs in favor of ROS-induced modifications, the result may be (enhanced) aging, “stress induced premature senescence” (SIPS)[26], malignant tumors[27,28] or even (neuro)degenerative diseases[29].

Damage of the “permanent structure” DNA is especially threatening to proliferating cells, since it can result in mutations (base substitution, deletions and strand fragmentation) and even carcinogenesis.

**Fig. 1:**
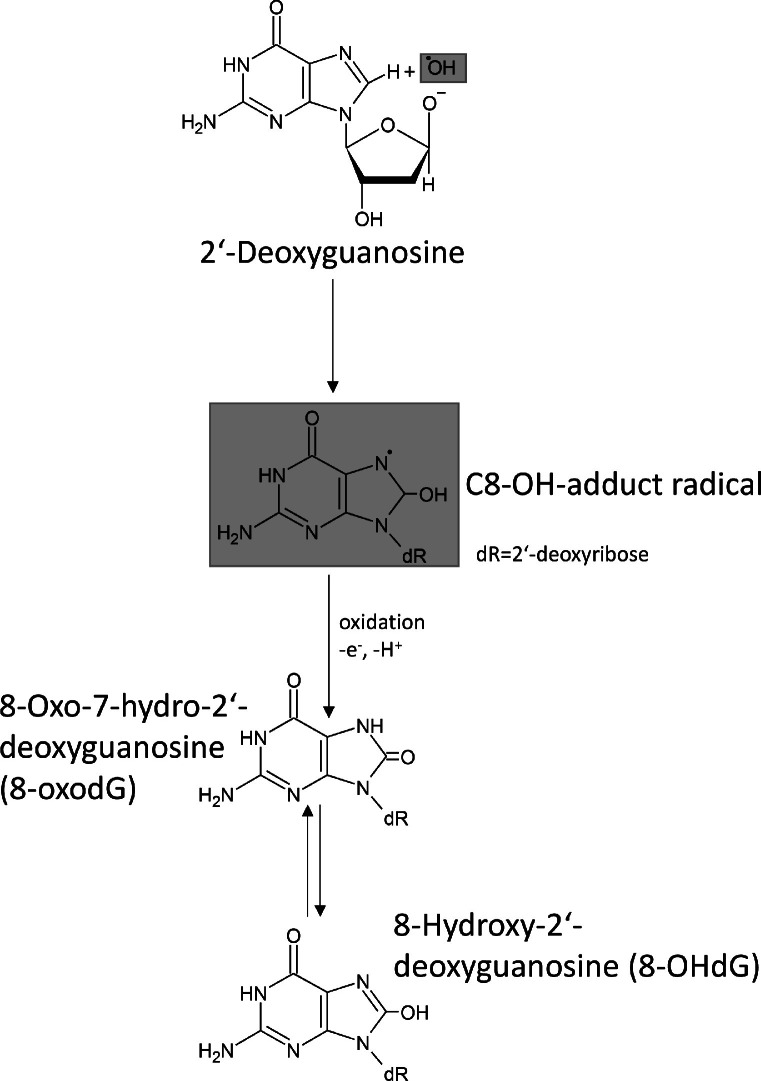
The reaction of 2-deoxyguanosine with a hydroxyl radical (upper part of this figure) forms amongst others the according radical adduct C8-OH. A subsequent oxidation results in 8-oxo-7-hydo-2′-deoxyguanosine (8-oxodG) or the according tautomer 8-hydoxy-2′-deoxyguanosine (8-OHdG, bottom part of this figure). In the literature, the terms 8-OHdG and 8-oxodG are often used for the same compound[69]. This figure is modified according to Valavanidis[30] et al.

Although oxidative damage to DNA produces a broad spectrum of reaction products, some of those products are highly specific (base and sugar modifications, covalent crosslinks, single- and double-strand breaks). One of the most frequently occurring lesions are 8‑hydroxy-2′-deoxyguanosine (8-OHdG) and 8-oxo-7-hydro-2‘-deoxyguanosine (8-oxodG)[30] (see Fig. 1), respectively, formed in vivo and can be both quantitatively and qualitatively detected in cells and tissues. One reason is that guanine is the most susceptible of all nucleobases to oxidative damage[31].

Since DNA damage plays a pivotal role in carcinogenesis, 8-OHdG has not only become a valid marker of oxidative DNA modification, but also an important one to asses cancer risk[30,[32], [33], [34], [35], [36]], as well as a predictor of prognosis in most solid tumors (via direct tumor sample, blood and urine)[37], [38], [39], [40], [41], [42], [43], [44], [45], [46], [47], [48]. A meta-analysis revealed a significant association between 8-OHdG formation and poor overall survival in cancer patients[49].

**Fig. 2:**
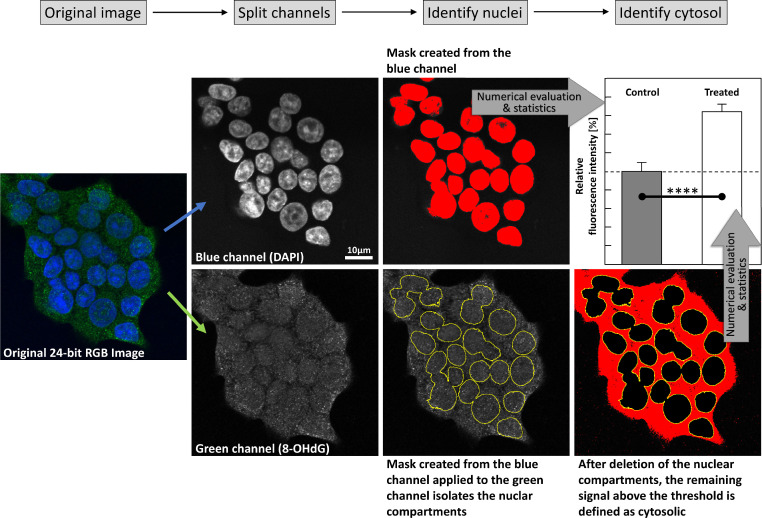
This image shows the typical workflow of an automated analysis of confocal microscopic images of immunolabelled cells and tissues. The original image (left, in this case clustered growing HCT116 cells) is split into its channels (here: Blue (DAPI-staining of the nuclear DNA) and Green (8-OHdG), second column); DAPI-staining is used to create a mask, that identifies and separates the nuclear compartments from the remaining sample (third column), while the cytosol is defined as “not nucleus, but brighter than the background” (last column). Intensity of the background was determined by the threshold applied, a limit selected by the user that truncates the signal below a certain limit (i.e. lowest cytosolic fluorescence intensity, in this case).

One of the most common detection methods for 8-OHdG is immunochemistry, paired with confocal/fluorescence microscopy, enabling high-resolution imaging of its intracellular distribution[50,51]. With extensive automation of high-volume microscopic image evaluation (see Fig. 2), high-throughput sample assessment can be performed.

However, in addition to the fluorescence intensity resulting from immunostained 8-OHdG, another effect is seen in the nucleus due to the corresponding DNA damage, namely the formation of so-called 8-OHdG foci, accumulations of this oxidative modification in highly condensed DNA, comparable to γ-H2AX foci[52] (formed in damaged DNA double strands, when H2AX is phosphorylated near the damage site) that can also be observed[53,54]. These foci serve as a signal to the cellular DNA repair system and aid in recruitment of repair proteins to the damage sites.

Consequently, altered patterns of 8-OHdG can be displayed within a sample, adding another parameter that helps to distinguish more clearly between healthy and possibly oxidatively damaged or even cancerous tissue, to which our own reliable 8-OHdG antibody, as demonstrated in this study, contributes.

This work aimed at production of an antibody that binds 8-OHdG in a highly specific manner, whose fluorescence signal correlates with the amount of nuclear 8-OHdG and which is able to detect the 8-OHdG foci comparable to a commercially available one.

## 2 Materials and methods

### 2.1 Antigen coupling and preparation

For mouse immunization and detection of antigen-specificity of secreted antibodies, 8-Hydroxy-2´-deoxyguanosine (8-OHdG, *Cayman Chemicals*, Tallinn, Estonia) was labeled by EDC-mediated crosslinking to hamster polyomavirus major capsid protein VP[55] and BSA, respectively. In order to select antigen-specific hybridoma cells 8-OHdG was coupled with Dylight633 via Conjugation Kit Fast Lightning-Link (*Abcam*, Cambridge, UK) according to manufacturer information.

### 2.2 Generation of anti-8-OHdG murine antibodies

C57BL/6 mice (9 weeks old) of the breeding colony at the University of Potsdam (Potsdam, Germany) were immunized with 70 µg 8-OHdG-VP in PBS by intraperitoneal injection on days 0, 7, and 14. The animal work was conducted according to relevant national and international guidelines. The study was approved by the Brandenburg Ministry of Environment, Health and Consumer Protection (reference number V3–2347-A16–4–2012). Seven days after third injection, blood samples have been taken and serum was used to verify the immune response by ELISA. Four days after the booster injection, the mouse was sacrificed, the spleen was removed and spleen cells were fused with transgenic myeloma cells according to Listek et al.[56]. Briefly, spleen/myeloma cell ratio was adjusted to 3:1 in 12.5 % PEG 8000 (*Sigma-Aldrich*, Munich, Germany) and electrofusion was performed using a voltage of 3500 V/cm for 20 μs. The fused cells were cultivated for 14 days in hypoxanthine-aminopterin-thymidine (HAT) supplemented RPMI full growth medium in three T75 culture flasks together with feeder cells under 6 % CO_2_, at 37 °C and 95 % humidity. Subsequently, the cells were collected and prepared for cell staining and sorting of antigen-specific antibody-producing cells.

### 2.3 Selection of antigen-specific antibody-producing hybridomas using selma

Fourteen days after fusion, antigen-specific screening was performed with hybridoma cells as previously described[56], [57], [58]. Before sorting, the presence of biotinylated artificial surface receptors on hybridoma cells was checked by flow cytometry using phycoerythrin-conjugated streptavidin (SAV, *Merck*, Darmstadt, Germany). Once proven, the antibody capture matrix was built by adding SAV-conjugated goat anti mouse IgG antibody (1 mg/ml) to hybridoma cells for 20 min at 4 °C. Afterwards, the cells were washed with 10 ml MACS buffer, the pellet was resuspended in 1 ml full growth cell culture media supplemented with 20 % (v/v) FCS, 2 mM glutamine, 50 μM ß-mercaptoethanol and the cells were incubated for 3 h at 37 °C and 6 % CO_2_ to allow antibody secretion and binding to the antibody capture matrix. Once again, cells were washed with 10 mL MACS buffer. The cell pellet was resuspended in 150 µL MACS buffer and 1.5 µL 8-OHdG conjugated to Dylight633 were added for 20 min at 4 °C. After a twice cell wash step with 10 ml MACS buffer, the pellet was resuspended in 500 µl MACS buffer supplemented with 3 µl 7-AAD (1 mg/ml, *ThermoFisher*, Waltham, MA, USA). The sample volume was adapted to 1 ml and 5 × 10^3^ cells were sorted in 24-well cell culture plates by flow cytometry (BD FACSAria™ III Cell Sorter, *Becton Dickinson*, Germany). The sorted antigen-specific hybrids were cloned by limiting dilution to obtain monoclonal hybridoma cells. Enzyme-linked immunosorbent assays were performed to select the most specific candidates.

### 2.4 Purification of murine anti 8-OHdG antibodies

Positively tested monoclonal hybridomas were cultivated in T75 culture flasks (*Greiner Bio-One*) to collect cell culture supernatant for antibody purification. The culture fluids were centrifuged (5000 g, 25 min, 4 °C), filtered (0.45 μm) and mixed in a 3:1 ratio with binding buffer (4 M NaCl, 2 M Glycin NaOH at pH=8.9), and were then passed over a protein A column (ProSep-vA Ultra Chromatography Media, *Millipore*, Schwalbach, Germany). The column was equilibrated and washed with wash buffer (binding buffer 1:3 diluted in H_2_O, 15 ml) and the bound antibodies were eluted using a pH=5 and a pH=3.5 elution buffer (0.1 M citrate) with a flow rate of 1 ml/min. The eluted antibodies were immediately neutralized with 500 μl 1 M Tris–HCl at pH=9.0 per 4.5 ml eluate. Purified antibodies were analyzed by means of SDS PAGE (12.5 %) as previously described[57].

### 2.5 IgG-subclass characterization of anti-8OHdG-antibody candidates

All candidates were characterized according to their murine IgG subclasses. Therefore, an enzyme-linked immunosorbent assay (ELISA) was performed. Briefly, microtiter plates (*Greiner-bio one*, Frickenhausen, Germany) were coated with 3 µg/ml goat anti-mouse IgG (Fc) antibody (*Jackson*) in PBS (50 µl/well) overnight at 4  °C in a humid chamber. The wells were washed three times with tap water and blocked with PBS/NCS (5 % neonatal calf serum, 50 µl/well) for 60 min at RT. After this, the wells were washed again and 5 µg/ml purified antibody candidates with 50 µl/well were added and incubated for 1 h at RT. After washing several secondary goat anti-mouse IgG-subclass specific antibodies conjugated to horseradish peroxidase (HRP; 1:5000, *Dianova GmbH*, Hamburg, Germany) were used for detection. The plates were incubated for 45 min at RT. Tetramethylbenzidine (TMB) solution (0.12 mg/ml TMB with 0.04 % hydrogen peroxide in 25 mM NaH_2_PO_4_) was used as substrate. The reaction was stopped after 5 min with 1 M H_2_SO_4_. Optical density (OD) was measured at 450 nm with a reference of 630 nm.

### 2.6 Immunostaining and preparation

As **primary antibodies**, a commercial one (Anti-8-Hydroxy-2′-deoxyguanosine antibody [N45.1-clone] from *Abcam*, #ab48508, monoclonal from mouse) was used (diluted 1:100 in PBS containing 5 % FCS) in order to compare the results to our own antibodies (see above).

As **secondary antibody**, AlexaFluor488 (*ThermoFisher*, #A-11,029, anti-mouse from goat) has been used, diluted 1:100 (in PBS containing 5 % FCS).

**DNase I** (*Roche*, #10,104,159,001, 0.5 mg/ml or 10 U/ml, respectively, prepared and applied according to the manual), **RNase A** (*Qiagen*, #1,018,048, 6.5 mg/ml, prepared and applied according to the manual).

**Washing buffer**: 5 % FBS (from *Gibco*) in PBS.

### 2.7 Cell culture

Human **FF95** dermal fibroblasts (obtained from human foreskin tissue of a 1-year old donor, kindly provided by Prof. Karin Scharffetter-Kochanek from the University of Ulm, Germany), **HT22** (immortalized mouse hippocampal neuronal line, provided by the Institute of Anatomy, Charite Berlin, Germany) and **HCT116** (human colorectal carcinoma line, purchased from the American Type Culture Collection (Rockville, MD)) cells are kept under growth conditions (37 °C, 5 % CO_2_) in uncoated T75 flasks (*ThermoFisher Scientific*, #156,499).

For treatment and fluorescence microscopy, cells were singled by trypsinization (1x Trypsin, *Bio&Sell*), seeded in poly-d-lysine-coated glass bottom petri dishes (*MatTek*, #P35GC-1.0–14-C) and kept under growth conditions overnight to ensure proper attachment of the cells.

Medium for FF95, HT22 and HCT116: high glucose DMEM (“DMEM/high glucose”, *Cytiva*, SH30243.01), containing GlutaMAX, 10 % heat-inactivated fetal bovine serum (*Gibco*), 1 % penicillin/streptomycin (from *Gibco*).

### 2.8 Induction of 8-OHdG in living cells

A stock solution of menadione (*Sigma*, #M5750) is freshly prepared (100 mM in PBS), the appropriate amounts of menadione (i.e. 20 µl for 1 mM) are added directly into the 2 ml of full medium covering the cells, according to Bilge Debelec-Butuner, et al.[53].

### 2.9 Acidic denaturation of DNA

For some commercial antibodies (such as the N45.1-clone used here), denaturation of the DNA appears to be critical in order to allow access of the antibody to the chromatin.

Denaturation was performed via incubation of the samples in 2 N HCl for 45 min at room temperature, followed by a neutralization step using either sodium tetraborate 0.1 M (pH=7.8, for 25 min) [59] or 50 mM Tris–HCl (pH=8.8, for 5 min) at RT [60].

### 2.10 Protocol for single cell immunostaining followed by fluorescence/confocal microscopy

- Seed cells on glass-bottom dishes, allow to attach overnight (incubator, 37 °C, 5 % CO_2_) in 2 ml of culture medium.
- *Optional*: incubation with 1 mM menadione in order to induce 8-OHdG (30 min, growth conditions).
- Discard medium, fix cells for 5 or 30 min at room temperature (RT) in 4 % paraformaldehyde (PFA) OR 1:1 methanol:acetone (15 min).
- Wash cells (3 × 5 min in washing buffer).
- Permeabilization: 5 min in 0.1 % TritonX-100 dissolved in PBS, ice cold.
- Wash cells (3 × 5 min in washing buffer).
- *Optional*: RNase A (1 h at RT)
- *Optional*: DNase I (1 h at RT)
- *Optional*: acidic denaturation of DNA (see above)
- Wash cells (3 × 5 min in washing buffer).
- Block non-specific binding sites (incubate 1 h in washing buffer at RT).
- Discard supernatant and incubate 1 h at RT with primary antibody (either N45.1, 1:100 diluted in washing buffer, or one of the produced clones, undiluted mouse serum).
- Wash cells (3 × 5 min in washing buffer).
- Incubate with secondary antibody (#A-11,029) diluted 1:100 in washing buffer.
- Wash cells (3 × 5 min in washing buffer).
- Prepare a permanent preparation with DAPI-containing mounting medium (ROTI®Mount FluorCare DAPI, #HP20.1, from *Roth*; 24 h drying time).

### 2.11 Blocking of the AF11-clone with its antigen to test its specificity

To test the specificity of the AF11-clone, the antibody was incubated for two hours with its target antigen 8-OHdG (*MadChemExpress*, Article# HY-W011540, “8-Hydroxy-2′-deoxyguanosine”, 10 mM, dissolved in 1 mL DMSO) according to [61].

Subsequently, 8-OHdG immunostaining was performed with the pre-incubated antibody in menadione-exposed FF95 fibroblasts to examine the reduction in binding capacity and thus the specificity of the AF11-clone.

### 2.12 Microscopy and image analysis via imagej macros

Both cells and tissues were examined via confocal microscopy (*Zeiss* LSM780, an inverse confocal laser scanning microscope, running standard software).

Image analysis was automatized using ImageJ-macros, individually adapted to the respective immunostained sample series. The typical workflow of image evaluation is depicted in Fig. 2. After splitting the 24-bit RGB-images into the three separate channels, the blue channel (DAPI-staining of the nuclear DNA) was used as mask to isolate the nuclear 8-OHdG signal (green channel). The signal above the threshold and outside of that mask was defined to be cytosolic, while the signal outside of the mask and below the threshold was defined as background.

**Fig. 3:**
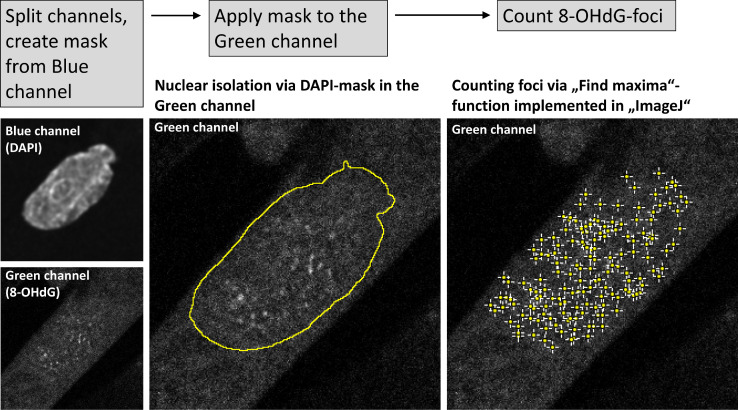
This figure depicts the workflow of nuclear 8-OHdG-foci counting using ImageJ. As in **Fig. 2**, the original image is split into the Blue and Green channel, a mask is created from the Blue one, applied to the Green channel and within the mask, foci are counted using the “*Find maxima*”-function[70] implemented in ImageJ. The amount of nuclear 8-OHdG-foci detected is divided by the nuclear area given in pixels, especially since the same objective without electronic post-magnification was used in all experiments.

Nuclear 8-OHdG foci have also been detected using an ImageJ macro (see Fig. 3 for details).

Threshold values required to exclude the background during image evaluation were set manually for a single image of the corresponding test series, and the remaining images were evaluated with the same threshold value. The corresponding channel masks (Fig. 2) were used to manually decide whether the applied threshold was suitable for all images.

### 2.13 Statistics

Experiments were conducted at least three times independently of each other, approximately 1000 cells were measured for each condition/treatment.

The numerical output of the ImageJ-macro was further processed and analyzed in GraphPad's “*Prism*” software (version 8.1.2).

First, data have been tested for Gaussian distribution (using the D'Agostino & Pearson, Anderson-Darling, Shapiro-Wilk and Kolmogorov-Smirnov tests implemented in “Prism”). According to the result, proper statistical tests were applied. For Gaussian distributed data: one-way ANOVA (Holm-Šídák) or Student's *t*-test (one-tailed, no correction), for not Gaussian data: one-tailed Student's *t*-test (Mann-Whitney) or non-parametric one-way ANOVA (Kruskal-Wallis), respectively. p-values < 0.05 were considered to be statistically significant.

The applied tests and significances are indicated in the according figures.

## 3 Results

### 3.1 Selection of the final antibody clone “AF11”

The antibodies produced in the course of this study were tested in HCT116 cells, treated with 1 mM menadione for 30 min and compared to an untreated control via confocal microscopy. Based on both automated evaluation and manual comparison, the single candidate “AF11” was finally selected. The individual evaluations of all 15 clones examined are compiled in Table 1. All further experiments were performed with that finally selected clone.

**Table 1:** All clones tested in HTC116 cells (1 mM menadione for 30 min.) and selection of the final candidate "AF11".

Clone	Intracellular distribution of 8-OHdG via confocal microscopy	Remarks
AA2	Manadione: almost no difference between nuclear and cytosolic 8-OHdG fluorescence, very few foci visible in nucleus, nucleoli sometimes very dark; control: nuclei darker than cytosol, few foci, mitochondria-like distribution of fluorescence in cytosol.	
AB12	Menadione: foci also in nucleus, but mainly in cytosol, nucleus darker than cytosol; control: nuclei very dark compared to cytosol, foci visible in both nucleus and cytosol	
BC1	Menadione: nuclei slightly darker than cytosol, no foci visible, slightly mitochondria-like distribution of fluorescence in cytosol; control: nuclei slightly darker than cytosol, foci visible in cytosolic volume	
BC7	Menadione: no difference between nucleus and cytosol, at times nucleus appears darker, foci randomly distributed; control: nuclei at times slightly darker than cytosol, foci very evenly distributed, yet more in cytosol.	
AF11	Menadione: strong “granular” nuclear signal; control: bright nuclei, clearly visible foci.	Final candidate
AE10	Menadione: dark nuclei, sometimes equal distribution of fluorescence between nucleus and cytosol, no foci; control: slightly darker nuclei until equal distribution of fluorescence, very few or no foci visible.	
AG8	Menadione: no difference between cytosol and nucleus, no foci; control: no foci, some nuclei appear slightly darker than cytosol.	
AH5	Menadione: no foci, “granular” nuclei, but little difference between nucleus and cytosol; control: nuclei darker than cytosol, no foci, fluorescence distribution looks more mitochondrial.	
AH9	Menadione: no difference between nucleus and cytosol in most cells, some nuclei are darker, foci are very evenly distributed; control: some nuclei are slightly, some significantly darker than cytosol, foci visible in nuclei.	
C12	Menadione: no difference between nuclei and cytosol, no foci; control: fluorescence evenly distributed, no foci, nuclei of larger islets sometimes darker.	
CF10	Menadione: nucleoli appear brighter than cytosol, no difference between nuclei and cytosol, no foci; control: no foci, bright nucleoli, nuclei sometimes darker than cytosol.	
CH2	Menadione: equal distribution of fluorescence in cytosol and nucleus, no foci; control: hardly any foci, homogeneous distribution of fluorescence between nucleus and cytosol.	
CH5	Menadione: no foci, no difference between nucleus and cytosol; control: nuclei slightly darker than cytosol, no foci, rather granular distribution of fluorescence.	
DD8	Menadione: even distribution in nucleus and cytosol, no foci; control: nuclei sometimes darker than cytosol with clearly visible foci.	
DF6	Menadione: no foci, nuclei appear slightly lighter than cytosol; control: nuclei darker than cytosol, foci visible in both compartments.	

### 3.2 Fluorescence intensity of immunolabelled 8-OHdG in different cell lines

**Fig. 4:**
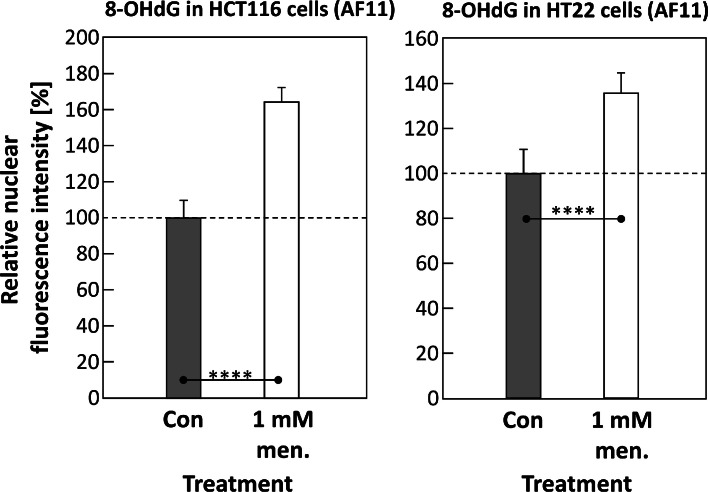
Here, the relative nuclear fluorescence intensity of immunostained 8-OHdG (“AF11”-clone) is depicted as control (Con, dark columns) compared to a sample, exposed to 1 mM of menadione for 30 min (light columns), in both HCT116 (left panel) and HT22 cells (right panel), respectively. The fluorescence intensity of the control has been defined as 100 %. Cells were cultivated, treated, fixed and analyzed as described in *Material and Methods*. Statistics: data passed normality-test, Student's *t*-test (one-tailed, no correction) applied; *: *p* ≤ 0.05; **: *p* ≤ 0.01; ***: *p* ≤ 0.001; ****: *p* ≤ 0.0001.

**Fig. 5:**
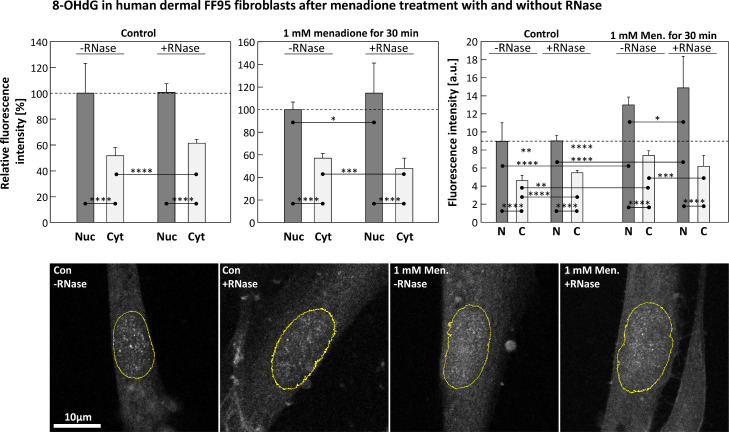
The upper row of panels shows the relative fluorescence intensities detected via confocal microscopy of immunostained 8-OHdG in FF95 cells. Left panel: nuclear (Nuc, dark column) and cytosolic (Cyt, light column) values of the untreated control, both with and without RNase treatment. Center panel: menadione exposed sample with and without RNase treatment. In both cases, the nuclear fluorescence intensity without RNase was defined as 100 %. The right panel depicts the according absolute fluorescence intensities for the nuclear (N) and cytosolic (C) compartments. The confocal microscopic images below show representative cells of the corresponding samples (Green channel, only), on which the yellow oval indicates the nucleus according to the DAPI channel (not shown). Statistics: data passed normality-test, ordinary one-way ANOVA applied; *: *p* ≤ 0.05; **: *p* ≤ 0.01; ***: *p* ≤ 0.001; ****: *p* ≤ 0.0001.

Both in HCT116 and HT22 cells, a significant increase of nuclear fluorescence intensity after menadione treatment (1 mM for 30 min) was detectable compared to an untreated control, as depicted in Fig. 4. The same result was observed in human dermal FF95 fibroblasts (Fig. 5, right panel, without RNase).

### 3.3 Impact of RNase and DNase I treatment

RNase treatment after fixation of the cells in order to remove possible unspecific binding sites increased the differences between nuclear and cytosolic fluorescence intensity (Fig 5, center and right panel) after 8-OHdG induction via menadione, resulting in a greater difference in fluorescence intensities between the two compartments nucleus and cytosol.

In contrast, RNase treatment of the unexposed control showed no improvement of the nuclear to cytosolic ratio of the measured fluorescence intensities (Fig 5., left panel).

**Fig. 6:**
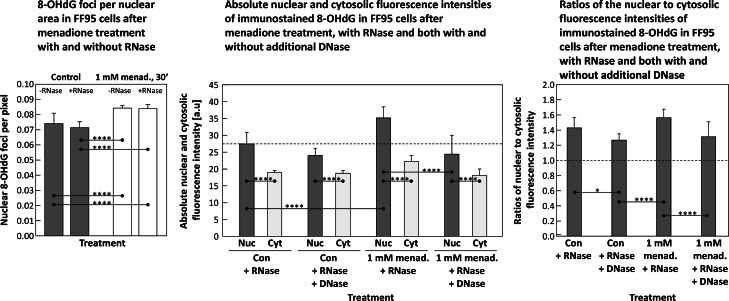
**Left panel:** nuclear 8-OHdG foci per area (in pixels) in FF95 cells of an untreated control (dark columns) compared to a menadione exposed sample (white columns), both with and without RNase. **Center panel:** absolute nuclear (Nuc) and cytosolic (Cyt) fluorescence intensities of immunostained 8-OHdG in FF95 dermal fibroblasts of an untreated control (Con) with and without DNase I compared to a menadione exposed sample (right part of this graph). All samples have also been incubated with RNase. Here are the absolute fluorescence intensities depicted, while the **right panel** displays the according nuclear to cytosolic intensity ratios. Statistics: data passed normality-test, ordinary one-way ANOVA applied; *: *p* ≤ 0.05; **: *p* ≤ 0.01; ***: *p* ≤ 0.001; ****: *p* ≤ 0.0001.

The amount of nuclear 8-OHdG-foci was determined and counted via an ImageJ macro (see Fig. 3 for details). Said 8-OHdG-foci were significantly increased after menadione exposure compared to an untreated control, but seems to be independently of RNase treatment if samples after same exposure to menadione are compared (i.e. control with and without RNase, as well as menadione exposed with and without RNase) (Fig. 6, left panel).

Treatment of the fixed samples with DNase I caused a significant reduction of the nuclear fluorescence intensity of both the untreated control and the menadione exposed sample (Fig. 6, **center panel**). Consequently, the ratio of nuclear to cytosolic fluorescence intensity of immunostained 8-OHdG was also significantly reduced (Fig. 6, right panel).

### 3.4 8-OHdG in human colon tissue

**Fig. 7:**
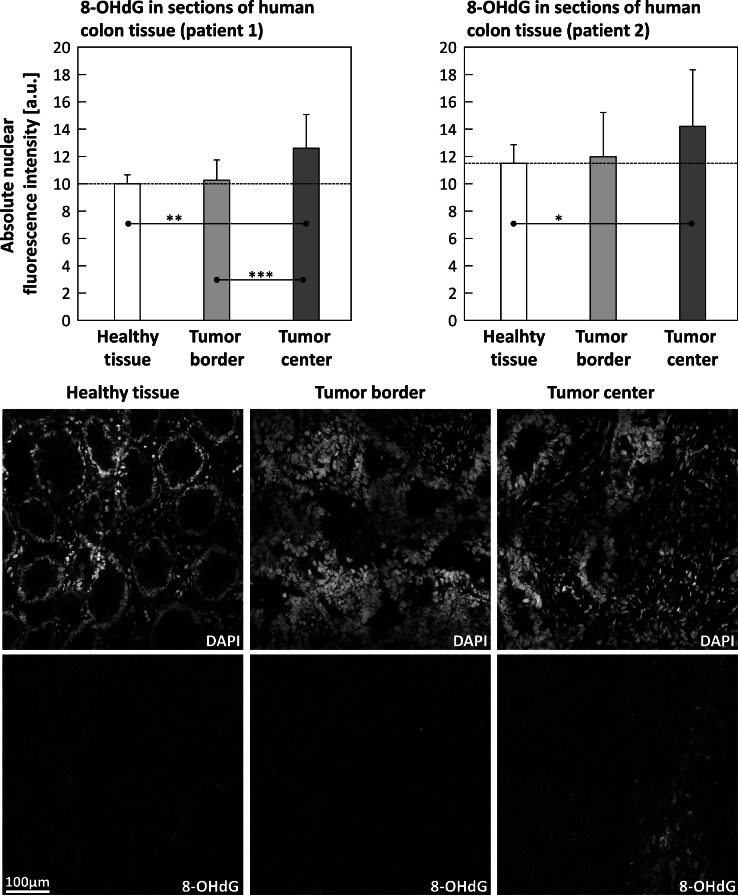
Upper part of this figure depicts the relative nuclear fluorescence intensities of immunostained 8-OHdG in colon tissue samples of two different cancer patients. Patient 1: male, 58 years old, left panel and patient 2: male, 65 years old, right panel. Healthy tissue (white columns) is compared to a sample from the tumor border (light grey columns) and from the tumor center (dark grey columns). The lower part displays representative confocal microscopic images of the corresponding tissue samples (patient 1 only). DAPI-staining is depicted in the upper row of images and the according 8-OHdG-channel in the bottom row. Statistics: data passed normality-test, ordinary one-way ANOVA applied; *: *p* ≤ 0.05; **: *p* ≤ 0.01; ***: *p* ≤ 0.001; ****: *p* ≤ 0.0001.

The comparison of three different samples (healthy colon tissue, samples from the tumor border, and samples from the tumor center) from two different patients (patient 1 (male, 58 years old, Fig. 7, left panel) and patient 2 (male, 65 years old, Fig. 7, right panel)) revealed the same trend in both: no difference between healthy tissue and tumor border, but a significant difference between healthy tissue and tumor center (both patients), as well as between tumor border and tumor center (patient 1, only).

### 3.5 The “AF11” 8-OHdG antibody compared to a commercial one

**Fig. 8:**
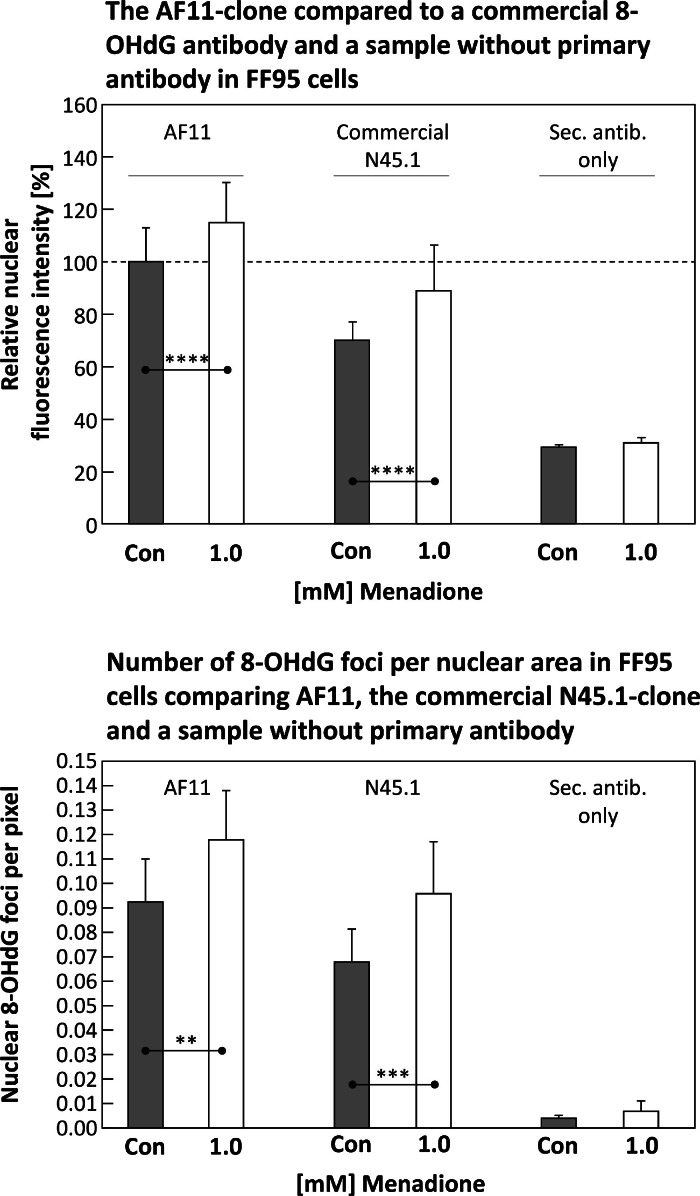
The upper panel compares the 8-OHdG antibody “AF11”-clone (left pair of columns, including an untreated control “Con” and menadione exposed cells) to a commercial one (*Abcam*, N45.1-clone, center pair) and samples incubated with the secondary antibody only (right) in FF95 cells. The lower panel presents the number of 8-OHdG foci per nuclear area given in pixels after the same treatment as above. All samples treated with RNase. Statistics: data passed normality-test, Student's *t*-test (one-tailed, no correction) applied; *: *p* ≤ 0.05; **: *p* ≤ 0.01; ***: *p* ≤ 0.001; ****: *p* ≤ 0.0001.

Compared to a commercial 8-OHdG antibody (*Abcam*, N45.1-clone), the clone “AF11” produced in the course of this work shows the same trend with respect to nuclear fluorescence intensity (confocal microscope), as shown in Fig. 8 (**upper panel**).

Both antibodies also show comparable trends regarding the measured 8-OHdG foci per nuclear area (Fig. 8, **lower panel**). Nonspecific binding of the secondary antibody (*ThermoFisher*, #A-11,029) used was found to be negligible (Fig. 8).

### 3.6 The effects of different fixation methods in FF95 fibroblasts

Different fixation methods showed a significant influence on the relative nuclear fluorescence intensities.

**Fig. 9:**
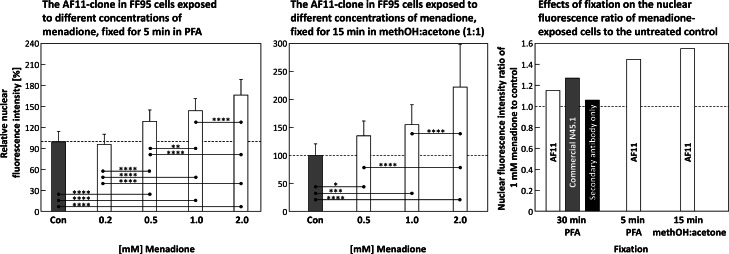
The **left panel** depicts the relative nuclear fluorescence intensity of immunostained (AF11-clone) 8-OHdG in FF95 cells exposed to different concentrations of menadione for 30 min, fixed for 5 min in paraformaldehyde (PFA). In the **center panel**, the same is depicted for FF95 cells fixed for 15 min in 1:1 methanol:acetone, while the **right panel** shows the according fluorescence intensity ratios of the nuclear 8-OHdG immunostaining (AF11) for cells exposed for 30 min to 1 mM of menadione compared to the according untreated control, for samples fixed for 30 min in PFA (left columns, the relative intensities are shown in **Fig. 8, upper panel**), 5 min in PFA (center column) and 15 min in 1:1 methanol:acetone (right column). Statistics: data passed normality-test, ordinary one-way ANOVA applied; *: *p* ≤ 0.05; **: *p* ≤ 0.01; ***: *p* ≤ 0.001; ****: *p* ≤ 0.0001.

The differences between menadione-exposed cells and the corresponding control were smallest in FF95 when fixed for 30 min in paraformadehyde (PFA, 4 %), as shown in Fig. 8. The according ratios are depicted in Fig. 9 (**left three columns of the right panel**). Shortening the PFA fixation to 5 min showed a significant increase in nuclear fluorescence (Fig. 9, **left and right panels**). Fixation with 1:1 methanol:acetone for 15 min showed the highest increase (Fig. 9, middle and right panels).

Comparing the results for HCT116 (Fig. 4, left panel) and HT22 (Fig. 4, right panel), the same menadione exposure and fixation (here: 1 mM menadione, then 30 min fixation in 4 % PFA) also show differences in the respective ratios of nuclear intensities (exposed and control), which are probably cell type-dependent.

### 3.7 Specificity of the AF11-clone

**Fig. 10:**
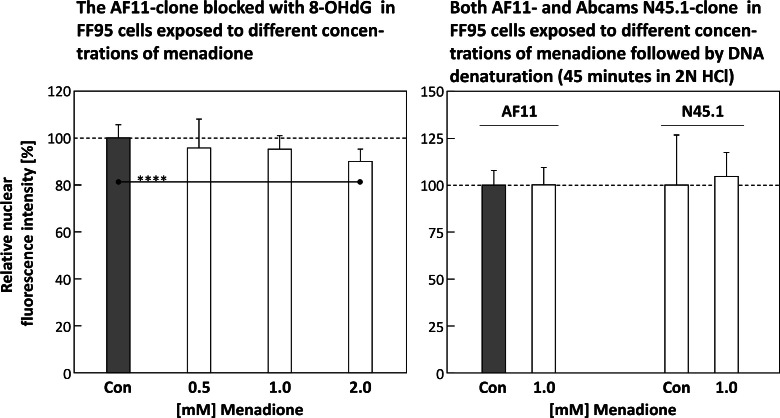
The **left panel** of this figure depicts the relative nuclear fluorescence intensities of immunostained 8-OHdG (AF11-clone), after blocking the primary antibody with its antigen 8-OHdG for 1 h, for cells exposed to different concentration of menadione for 30 min. In the **right panel**, the effects of DNA-denaturation (45 min in 2 N HCl) are depicted for both AF11 (left pair of columns) and the commercial N45.1-clone (right pair) in cells exposed to 1 mM of menadione for 30 min compared to an untreated control. In both panels, the samples were fixed in 4 % PFA for 5 Min. Statistics: data passed normality-test, ordinary one-way ANOVA and Student's *t*-test (one-tailed, no correction, right panel) applied; *: *p* ≤ 0.05; **: *p* ≤ 0.01; ***: *p* ≤ 0.001; ****: *p* ≤ 0.0001.

Pre-incubation of the AF11-clone with 8-OHdG for 2 h significantly reduced its binding capacity to menadione-induced 8-OHdG in FF95 fibroblasts (Fig. 10, left panel). There was no statistically significant difference in the nuclear fluorescence intensity between control and cells treated with 0.5 and 1.0 mM menadione for 30 min. After incubation with 2.0 mM menadione, there was even a significant reduction in nuclear fluorescence intensity.

The 8-OHdG for pre-incubation of AF11 was dissolved in DMSO, but incubation of the antibody with the corresponding concentration of the solvent only (here: 3 % DMSO) revealed no significant reduction in its binding capacity (data not shown).

### 3.8 DNA-denaturation via 2 N HCl

Acidic unfolding of DNA massively reduced the binding capacity of both AF11 and the commercial clone N45.1 to nuclear 8-OHdG in FF95 cells (Fig. 10, right panel). A significant difference between untreated control and cells incubated with 1 mM menadione was not detectable.

## 4 Discussion

The main objective of this study was to produce an 8-OHdG-specific antibody that can detect the corresponding oxidative DNA modification both qualitatively and quantitatively by automated evaluation of fluorescence microscopic images. This goal was achieved in that significant differences were found between untreated control cells and samples whose DNA was damaged with menadione for both nuclear amount of 8-OHdG and formation of so-called 8-OHdG foci (Fig. 4, Fig. 5, Fig. 6). The newly created antibody was also shown to be absolutely comparable to a commercially available one (Fig. 8).

Another aspect of the application is also the reliable differentiation between tumor tissue and healthy tissue. However, in the tissue samples it was only possible to distinguish both healthy tissue and the tumor margin from the tumor core. In contrast, differentiation between healthy tissue and the tumor border was not possible (Fig. 7). This problem could be solved in future studies if 8-OHdG is colocalized with other markers of oxidative DNA damage such as γ-H2AX and 53BP1.

Extensive automation of image analysis of fluorescence microscopic data using ImageJ-macros[62] also proved to be a viable method for rapid mass analysis of data. Partitioning of an image into nuclear, cytosolic, and background compartments using the DAPI channel can be robustly automated, as can the counting of foci within nuclei. The same parameters are also provided by the ALKIDES system.

Also of interest was the relatively small but significant decrease in the nuclear fluorescence signal after DNase I treatment (Fig. 6). This could be due to the fact that histones represent a stearic barrier for DNase I digestion.

In genetics, regions of chromatin that are sensitive to DNase I cleavage are referred to as “DNase I hypersensitive sites” (DHS)[63]. In these regions, chromatin has lost its condensed structure, exposing DNA and making it accessible for degradation by enzymes like DNase I – this is usually accompanied with increased transcriptional activity. In resting fibroblasts, on the other hand, only a small number of DNase I hypersensitive sites are found[64].

Consequently, DNase I treatment is unable to massively degrade nuclear DNA that is still condensed and associated with histones. Such a degradation would actually cause elimination of binding sites for an 8-OHdG antibody. More specifically, “binding site” here means an 8-OHdG modification that is still bound to nuclear DNA and cannot be washed out after incubation with the primary antibody. This also explains the still visible and sharply defined fluorescence microscopic DAPI signal.

In contrast, the increased ratio of nuclear to cytosolic fluorescence after RNase treatment of the samples actually appears to be due to the removal of nonspecific RNA binding sites in the cytosol (Fig. 5).

Different fixation methods tested (Fig. 9) showed clear differences between cells of the same line (here: FF95), but should also be adapted if necessary depending on the cell line to be examined.

The reason why acidic denaturation of the DNA in FF95 cells led to a complete loss of substrate recognition in both AF11 and N45.1 (Fig. 10, right panel) must be clarified in follow-up experiments, including other cell lines.

8-OHdG-specificity of AF11 appears to be sufficiently high, as also binding to the antigen, especially since AF11 pre-incubated with 8-OHdG was no longer able to bind significant amounts of nuclear 8-OHdG in FF95 cells (Fig. 10, left panel).

## 5 Conclusion

The here presented data demonstrates that the new antibody detects specifically 8-OHdG in a sensitive and highly specific manner. In the long term, this antibody will become part of a commercial kit available for the ALKIDES system (https://www.medipan.de/products-category/systems/) that will allow almost completely automated sample evaluation, and likewise local visual cell assignment in histological specimens, in contrast to analytical methods such as ELISA, HPLC, GC–MS[65]. This enables at the same time fully automated high-throughput processing and result assessment for multiple patients or entire cohort studies, as well as simultaneous measurement and colocalization of different biomarkers (such as γ-H2AX and 53BP1[66,67], the p53-binding protein 1, a crucial component of DNA double-strand break signaling and repair in mammalian cells) at the same time at cellular level [68] – allowing simple and effective visualization of DNA damage in oncological or pathological questions.

## Funding

The publication was funded by "Programm zur Förderung von Forschung, Innovationen und Technologien" (ProFIT Brandenburg). Open Access funding enabled and organized by Projekt DEAL. The publication of this article was funded by the Open Access Fund of the Leibniz Association. Tobias Jung was supported by DZD ("German Center for Diabetes Research", "Grant 82DZD0034G").

## CRediT authorship contribution statement

**Tobias Jung:** Writing – original draft, Investigation. **Nicole Findik:** Resources. **Bianca Hartmann:** Resources. **Katja Hanack:** Resources, Investigation. **Kai Grossmann:** Resources. **Dirk Roggenbuck:** Project administration. **Marc Wegmann:** Methodology. **René Mantke:** Project administration. **Markus Deckert:** Project administration. **Tilman Grune:** Writing – review & editing, Project administration.

## Declaration of competing interest

Dirk Roggenbuck is an employee of and owns shares in GA Generic Assays and Medipan GmbH, diagnostic manufacturers. Marc Wegmann is an employee of GA Generic Assays and Medipan GmbH, diagnostic manufacturers.

## Data Availability

Data will be made available on request.
